# Destructive coastal sea level oscillations generated by Typhoon Maysak in the Sea of Japan in September 2020

**DOI:** 10.1038/s41598-022-12189-2

**Published:** 2022-05-19

**Authors:** Igor P. Medvedev, Alexander B. Rabinovich, Jadranka Šepić

**Affiliations:** 1 Shirshov Institute of Oceanology, Russian Academy of Sciences, Moscow, Russia; 2 Moscow Institute of Physics and Technology, Dolgoprudny, Russia; 3 Institute of Ocean Sciences, Department of Fisheries and Oceans, Sidney, BC Canada; 4 Faculty of Science, University of Split, Split, Croatia

**Keywords:** Physical oceanography, Natural hazards, Ocean sciences

## Abstract

Typhoon *Maysak* (*Julian* in the Philippines) was a powerful tropical cyclone that strongly impacted coastal regions of the Sea of Japan on 2–4 September 2020. Destructive winds, violent storm waves, and intense rainfall occurred in Japan, on the Korean Peninsula, and in Far-Eastern Russia. Devastating coastal floods caused severe damage to coastal infrastructure and to ships and boats anchored in harbours and were responsible for numerous deaths. Our study indicates that the main reason for the destructive floods was the superposition of storm surge, extreme seiches (meteorological tsunamis), and surf beats. At various sites, different types of sea level oscillations prevailed depending on the atmospheric forcing, local topographic properties, and resonant shelf/coastal zone features. The principal forcing factors of these oscillations were atmospheric pressure and wind stress, but the exact generation mechanism of each specific type of oscillation was strongly site dependent. The uniqueness of the sea level response at each site is the main challenge in our understanding of the generation process and to the mitigation of the hazardous consequences of possible future events.

## Introduction

Tropical cyclones (typhoons and hurricanes) are among the most dangerous natural hazards that can result in major damage and considerable loss of life^1^. The eastern coasts of countries bordering the northwestern Pacific Ocean, such as the Philippines, Japan, North and South Korea, Russia and China, are regularly impacted by devastating typhoons. In September 1959, Typhoon Vera caused catastrophic damage and killed more than 5000 people on the coast of Japan, becoming the strongest and deadliest recorded typhoon to hit this region. Typhoon Nina in August 1975 severely damaged parts of China and killed several tens of thousands of people^1^. In August 1981, Typhoon Phyllis struck the coasts of North Japan, Sakhalin Island and the Russian Far East mainland, destroying infrastructure and producing deadly floods^2^.

Typhoon-generated coastal floods were likely the main factor responsible for the major destruction and high death toll during the above events. Recent studies based on extensive observations further indicate that typhoon-related flood effects are strongly site-dependent: major floods and associated intense currents can occur at some sites but have a minor effect at neighboring sites^3^. Such effects were observed during Typhoon Maysak, which in September 2020 destructively affected the coasts of Japan, South Korea, North Korea, and Russia. The present study uses satellite, hydrometeorological and tide gauge data to examine the sea level response to Typhoon Maysak forcing, including the physical properties and generation mechanisms of the sea level oscillations and the effects of local topographic resonance on the corresponding flood features.

## Typhoon Maysak

**Figure 1:**
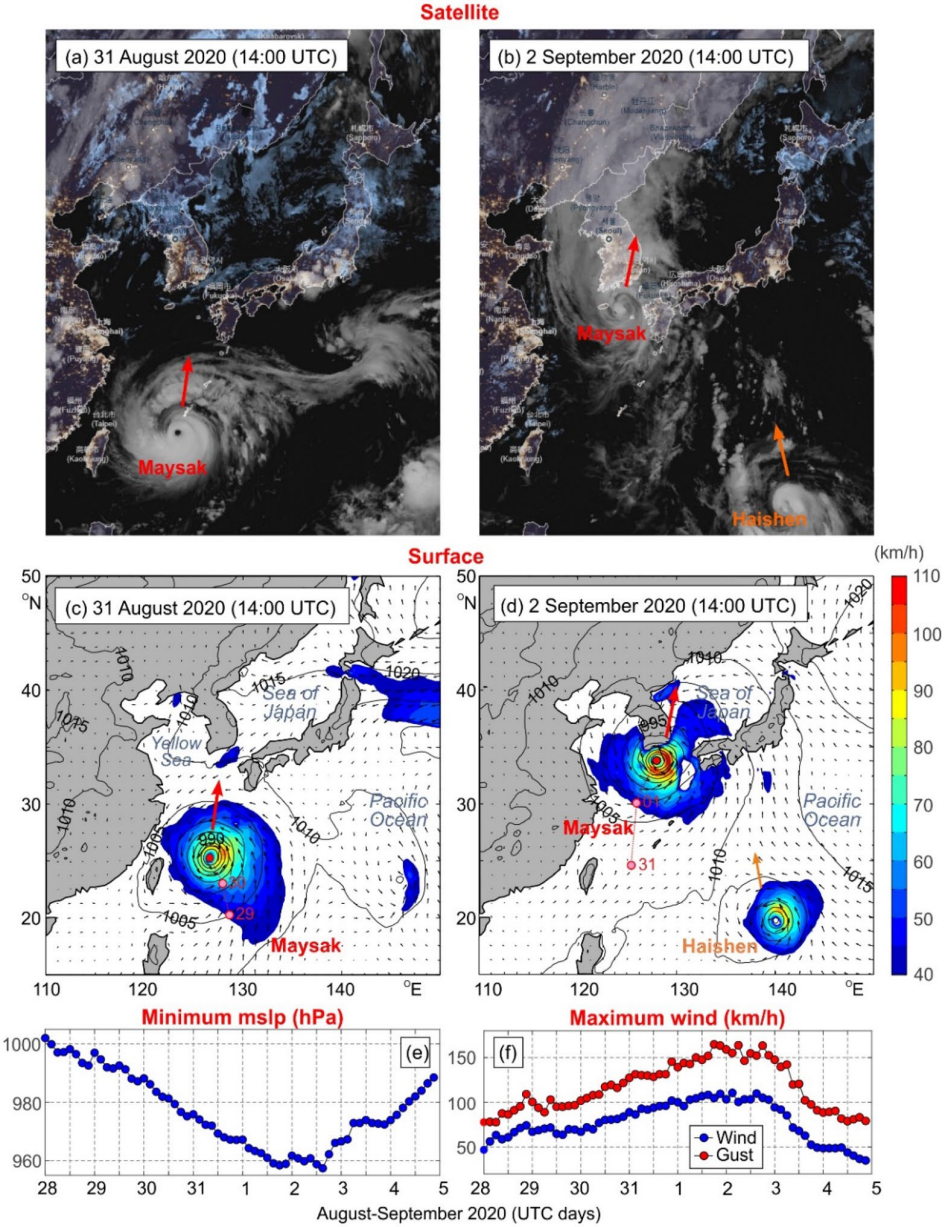
Satellite images of typhoon Maysak on (**a**) 31 August and (**b**) 2 September 2020; the thick red arrows show the direction of the typhoon propagation. The surface air pressure (hPa) and wind velocity (km/h) maps during typhoon Maysak for the east coast of Asia on (**c**) 31 August and (**d**) 2 September 2020 (from ERA5 Reanalysis); only wind speeds greater than 40 km/h are coloured. The red and pink circles show the typhoon center at current and previous dates, respectively; in the right bottom corners of (**b**) and (**d**) typhoon Haishen is shown to appear. ERA5 values of Maysak-related (**e**) minimum mean sea level air pressure (MSLP) and (**f**) maximum 10-m wind speed (blue) and wind gust (red). Satellite images were downloaded from ventusky.com. Maps were created with MATLAB 2021. Subplots were combined and edited with CorelDraw 2021.

Typhoon Maysak began its long-lived journey as a stationary depression formed over the western part of the Pacific Ocean east of the Philippines on 27 August 2020. On 28 August, the Philippine Atmospheric, Geophysical, and Astronomical Services Administration (PAGASA) assessed the system as a “tropical depression” and gave it the local name *Julian*^4^. At 06:00 UTC of the same day, the Japan Meteorological Agency (JMA) judged that the depression had strengthened into a “tropical storm” and assigned it the name *Maysak.* At 18:00 UTC, the JMA evaluated that the depression had further intensified to a severe tropical storm^5^. According to the JMA, Maysak had become a typhoon by 12:00 UTC on 29 August after developing “an eye”; at this time the maximum 10-min sustained surface winds reached 120 km/h, and wind gusts 175 km/h^6^ (Fig. 1).

Moving northward, Typhoon Maysak strongly affected the coasts of the Korean Peninsula, Russia, and Japan. A Panamanian-registered carrier “*Gulf Livestock 1*” sank ~ 100 miles west of Amami Ōshima Island (Japan) on 2 September 2020 due to this typhoon; only two of the 43 crew members were rescued. Two fatalities were reported in South Korea and several people were killed in North Korea, where major damage was observed. In Ulysses Bay in Vladivostok, a floating dock, which was blown away by the wind, eventually destroyed several boats. The floating crane “*Chernomorets-34*” ran aground and two crew members were killed. In total, the typhoon caused more than 40 deaths, damaged more than 9000 houses and over 250,000 customers lost power in South Korea and Russia. Total economic losses exceeded $100 million.

We analysed typhoon properties and propagation path in more detail using ERA5 Reanalysis data. Strong intensification of Typhoon Maysak began by 12:00 UTC of 29 August, when it started to steadily propagate towards the north (Fig. 1a–d). According to the ERA5 data, during the following 48 h, mean sea level air pressure (MSLP) at the typhoon eye decreased from 992.6 hPa (12:00 UTC of 29 August) to 969.3 hPa (12:00 UTC of 31 August) (Fig. 1e), the maximum hourly 10-m wind speed increased from 65 to 94 km/h and the maximum 3-s wind gusts reached 129 km/h (Fig. 1f). Within the next 24 h, the typhoon further strengthened, with the minimum MSLP dropping up to 961.0 hPa by 15:00 UTC of 1 September, while the maximum hourly wind speed increased up to 107 km/h and wind gust up to 152 km/h. Afterwards, Typhoon Maysak began to veer slightly towards the west, but kept its dominant northward propagation component—thus setting on the path across southern Japan and the Korea Strait. The typhoon mostly kept its strength during the following 24 h reaching hourly wind speed of 111 km/h and wind gusts of 165 km/h by 03:00 UTC of 2 September and minimum MSLP of 957.4 hPa at 15:00 UTC of the same day (Fig. 1). By this time typhoon increased its translational speed, and strongly affected the southern coast of the Korean Peninsula, and South Korea’s second largest city, Busan. Following arrival at the Korean Peninsula, the typhoon weakened, turning into a Category 3 system, and sped up towards the north crossing briefly over the westernmost sector of the Sea of Japan, hitting the mainland close to the North Korea, China, and Russia border around 3:00 UTC on 3 September (Fig. 3). The minimum MSLP was still rather low (967.3 hPa), and wind strong: the maximum hourly wind speed was up to 92 km/h, and the maximum 3-s wind gusts were up to 140 km/h. From this point on, Typhoon Maysak swiftly derogated and transitioned into an extratropical low, which continued to propagate northwestward across China. However, a few days after Typhoon Maysak had transited northward, another destructive super typhoon—Typhoon Haishen (https://blogs.nasa.gov/hurricanes/tag/haishen-2020/) – began to impact the region, demonstrating once again the threat that typhoons bring to coastal areas of the Sea of Japan. The onset and trajectory of Typhoon Haishen is presented in Fig. 1b,d.

### Atmospheric processes

**Figure 2:**
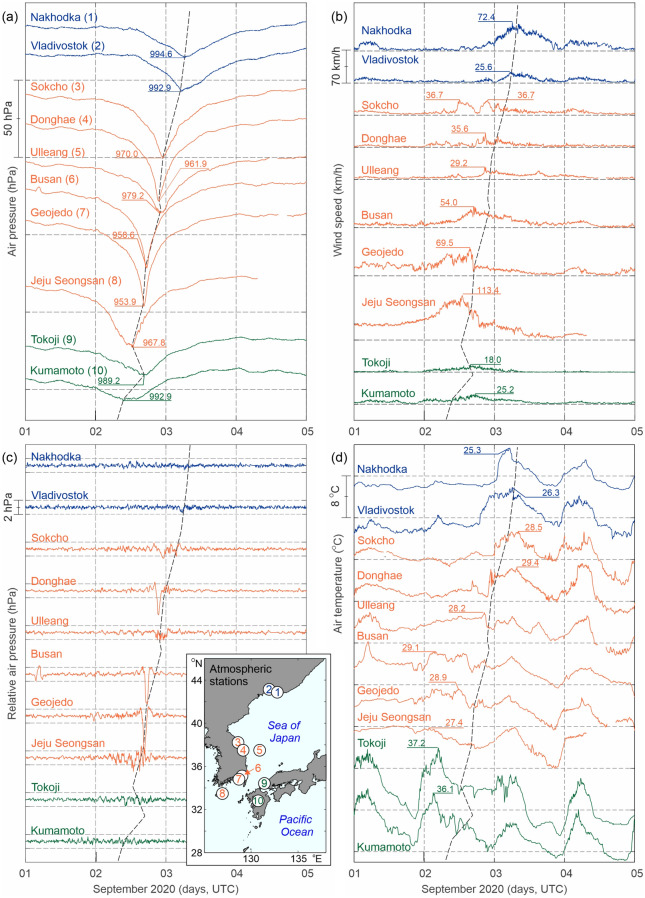
(**a**) Air pressure (hPa), (**b**) wind speed (km/h), (**c**) high-pass filtered air pressure (hPa) records using a 3-h Kaiser–Bessel window, and (**d**) air temperature (°C) records at ten weather stations for 1–5 September 2020. Locations of the stations (indicated by numbers 1, 2,…, 10) are shown in the inset in (**c**); the names of the weather stations are shown in plot (**a**). For all plots and the inset, records and circles in blue denote Russian stations, orange South Korean stations and green Japanese stations. Map in inset was created with MATLAB 2021. Subplots were combined and edited with CorelDraw 2021.

Air pressure time series measured at Japanese, South Korean and Russian stations (Fig. 2a) clearly depict northward propagation of Typhoon Maysak. It was deepest over the South Korean Island of Jeju (station Jeju Seongsan No. 8 in the inset in Fig. 2c), and over the coastal cities of Busan (No.6) and Geojedo (No. 7). The Geojedo air pressure measurements are especially remarkable: air pressure dropped for 50 hPa within 16.5 h, and for the final 25 hPa within just 2.5 h, reaching a minimum value of 953.9 hPa at 16:30 UTC of 2 September 2020. Some 350 km to the east of the main typhoon track, i.e. over the Japanese towns of Tokoji (No.9) and Kumamoto (No. 10), the typhoon was much weaker: the minimum air pressure values dropped to 989.2 hPa. Weakening of the typhoon as it moved northward is also evident from the depicted time series: closer to the northern border of South Korea the minimum air pressure values were ~ 970 hPa (stations No. 3–5), and at Far-Eastern Russian cities of Nakhodka (No. 1) and Vladivostok (No.2) the minimum air pressure values were just above 990 hPa (Fig. 2a). Wind measurements reflect similar features associated with the typhoon propagation track (Fig. 2b): the strongest winds were measured over three South Korean locations (Jeju Seongsan, Busan, and Geojedo). There are, however, some differences with respect to the air pressure: the strongest winds, reaching 113.4 km/h, were recorded at the island station Jeju Seongsan, whereas the recorded winds at Busan and Geojedo were weaker: 54.0, and 69.5 km/h, respectively. A peculiar feature of wind time series is an abrupt drop in wind speed at Geojedo—this drop coincides with timing of the minimum air pressure at this station—implying that the calm “eye” of typhoon crossed over the city at this moment. Winds attenuated as the typhoon moved northward. Nonetheless, at Nakhodka 72.4 km/h winds were recorded.

Marked high-frequency air pressure (AP) fluctuations were observed at many stations along the coasts of Japan, South Korea, and Russia (Fig. 2c). The strongest AP fluctuations were recorded over the South Korean stations Jeju Seongsan, Geojedo, Busan, and Donghae (No. 4). Clearly, the main aspect of these features is a pronounced pressure drop associated with the arrival of the typhoon centre. The estimated typhoon propagation speed between Geojedo/Busan and Donghae is ~ 60 km/h. The distinct air pressure drop was preceded and followed by multiple shorter-period fluctuations with ranges of up to 2–4 hPa evident at most of the Korean stations. However, such disturbances are not seen at most of the Japanese and Russian stations (Fig. 2c). Typhoon Maysak was also detected in the air temperature series (Fig. 2d). Depending on the station location, the arrival of the warm tropical air mass to the area is recorded either before arrival of the typhoon centre (at the Japanese stations and the Korean stations to the south of Ulleang) or simultaneous/shortly after arrival of the typhoon centre (at the two northernmost Korean stations and Russian stations). The typhoon is evident over the four northernmost stations, where typhoon-advected warm air had much higher temperatures (up to 8 °C) than the air previously residing over the area, and where the typhoon-related range of temperature variations strongly surpassed that of daily temperature variations. In general, the four plots shown in Fig. 2 clearly demonstrate that air pressure, wind and air temperature were highly correlated.

## Results

### Sea level oscillations

**Figure 3:**
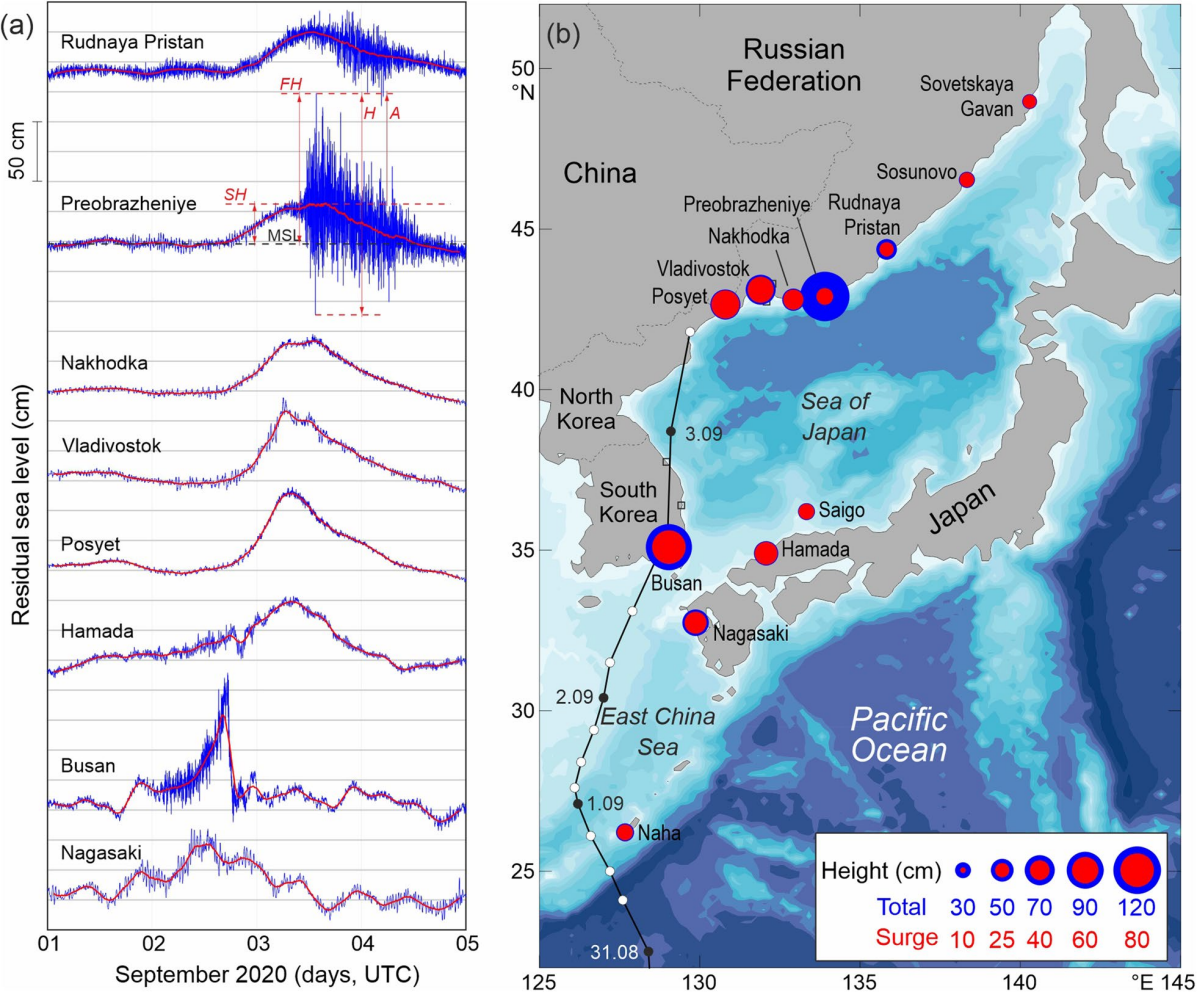
(**a**) Residual (de-tided) sea level records at eight stations during the passage of Typhoon Maysak; the thick solid red curves show the low-frequency component associated with the storm surge; (**b**) the September 2020 track of Typhoon Maysak and locations of the tide gauges used in this study; the circle size corresponds to the total flood height (blue) and surge height (red) above the mean sea level. The maximum observed flood height (*FH*), surge height (*SH*), amplitude (*A*) and height (*H*) of seiches/IG-waves are illustrated for the sea level curve at Preobrazheniye. Map was created with MATLAB 2021 using GEBCO bathymetry product. Subplots were combined and edited with CorelDraw 2021.

This study examines the hazardous sea level oscillations and coastal floods that impact the Sea of Japan. Analyses are based on data from four Japanese, one South Korean, and seven Russian tide gauges (Table 1; Fig. 3). The sources of the data, their main properties and methods used to examine these data are described in “Data and methods”. Figure 3a shows the residual records for eight stations located along the track of Typhoon Maysak. Two types of sea level oscillations are evident in these records: (1) Low-frequency variations with durations of ~ 2 days; and (2) high-frequency oscillations superimposed on the low-frequency displacements. The first type of sea level change is caused by storm surges produced by the combined effect of falling air pressure and intense onshore winds^7^. The second type has periods from minutes to hours and is associated with storm-generated seiches^8^ and infragravity (IG) waves (surf beats) produced by nonlinear interaction of the wind waves^8,9^.

**Table 1:** List of tide gauges and observed surge and seiche (including IG-waves) parameters for the corresponding records during the 2020 Typhoon Maysak on the coasts of Japan, South Korea, and Russia. “Flood height” (FH) is the absolute displacement height relative to the mean sea level (MSL); “surge height” (SH) is the height of the storm surge solely above the MSL. The approach for the estimation of these parameters is shown in Fig. 3a for Preobrazheniye station. Location of the stations is shown in Fig. 3b.

Station	Country	Coordinates		Flood height (cm)	Surge height (cm)	Seiches and IG-waves		
		Lat (°N)	Long (°E)			Amplitude (cm)	Height (cm)	Period (min)
Naha	Japan	26.21	127.67	36	29	6.3	17	26, 4
Nagasaki	Japan	32.74	129.87	59	47	13	34	37
Hamada	Japan	34.90	132.07	53	49	4.5	22	17
Saigo	Japan	36.20	133.33	33	29	3.7	15	32, 18, 7
Busan	South Korea	35.09	129.04	115	79	36	85	52, 16, 6.5
Posyet	Russia	42.65	130.80	70	64	5.2	9.3	30, 17, 9
Vladivostok	Russia	43.11	131.90	69	58	11	20	39
Nakhodka	Russia	42.80	132.92	48	42	5.7	11	36, 8.5
Preobrazheniye	Russia	42.90	133.90	124	32	92	185	35
Rudnaya Pristan	Russia	44.37	135.83	43	25	18	68	14
Sosunovo	Russia	46.53	138.33	30	26	4.3	17	53
Sovetskaya Gavan	Russia	48.97	140.29	27	23	3.6	13	21

One of the most intriguing features of the records is their strong individuality: the sea level response to the moving typhoon at each site is unique to that site. The striking difference is apparent both in the total flood heights and in the composition of the recorded oscillations, whereby, the relative contribution of each component—storm surge, seiches, and IG-waves—was site-specific. In particular, IG-waves strongly prevailed at the Russian stations of Preobrazheniye and Rudnaya Pristan, while storm surge was especially strong at Posyet and Busan. The latter stations were also impacted by intense seiches. Marked seiches were further observed at Nagasaki and Hamada. The cumulative effect of IG-waves and seiches enhanced the flood impact on the affected coasts.

To separate storm surge and high-frequency SL oscillations, we high-pass filtered the data with a 3-h Kaiser–Bessel window^10^. The red curve in Fig. 3a delineates the low-frequency (storm surge) component of the recorded SL changes. Being phase-locked to Typhoon Maysak, the storm surge moved in the northward direction. In the East China Sea, the surge reached its maximum height of 29 cm at Naha and 47 cm at Nagasaki at 10:00 UTC on 2 September. At Busan, a maximum surge of 79 cm was observed at 16:00 UTC of the same day. The surge at Busan had a very specific shape, with a fast (much faster than at other stations) but gradual SL raise followed by an abrupt fall of ~ 90 cm in about 70 min. This effect was found to have been due to the rapid change in the wind direction, from onshore to offshore after the typhoon center passed (not shown for brevity). On the Russian coast, the storm surge peak occurred at 10:00–12:00 UTC on 3 September, reaching a height of 58 cm at Vladivostok and 64 cm at Posyet. In the northern part of the Sea of Japan (sites Rudnaya Pristan, Sosunovo, and Sovetskaya Gavan), located at the periphery of the typhoon, the maximum surge heights were < 26 cm (Fig. 3b, Table 1).

The character of the high-frequency SL oscillations was markedly different at different sites. At Nagasaki, the trough-to-crest height was 34 cm, at Hamada 22 cm, and at Vladivostok 20 cm (Fig. 3a). In all cases, the oscillations were formed during the rising stage of the surge. Exceptional high-frequency (HF) sea-level variations were observed at Busan. Here, the variations began to intensify as soon as the storm surge began and reached a maximum height of about 85 cm at the time of the sharply falling surge; the cumulative effect of the storm surge and high-frequency oscillations caused hazardous flooding of 115 cm at this station (Table 1). The strongest HF oscillations were observed at Russian stations Preobrazheniye and Rudnaya Pristan (Fig. 3a,b). These oscillations at the two stations had much higher frequencies than those at Nagasaki, Hamada, Busan and Vladivostok and appear to be related to IG-waves associated with extremely high storm waves. The heights of these oscillations dramatically increased just after the peak of the storm surge. The maximum trough-to-crest height of the HF component at these stations reached 68 cm at Rudnaya Pristan and 185 cm at Preobrazheniye.

**Figure 4:**
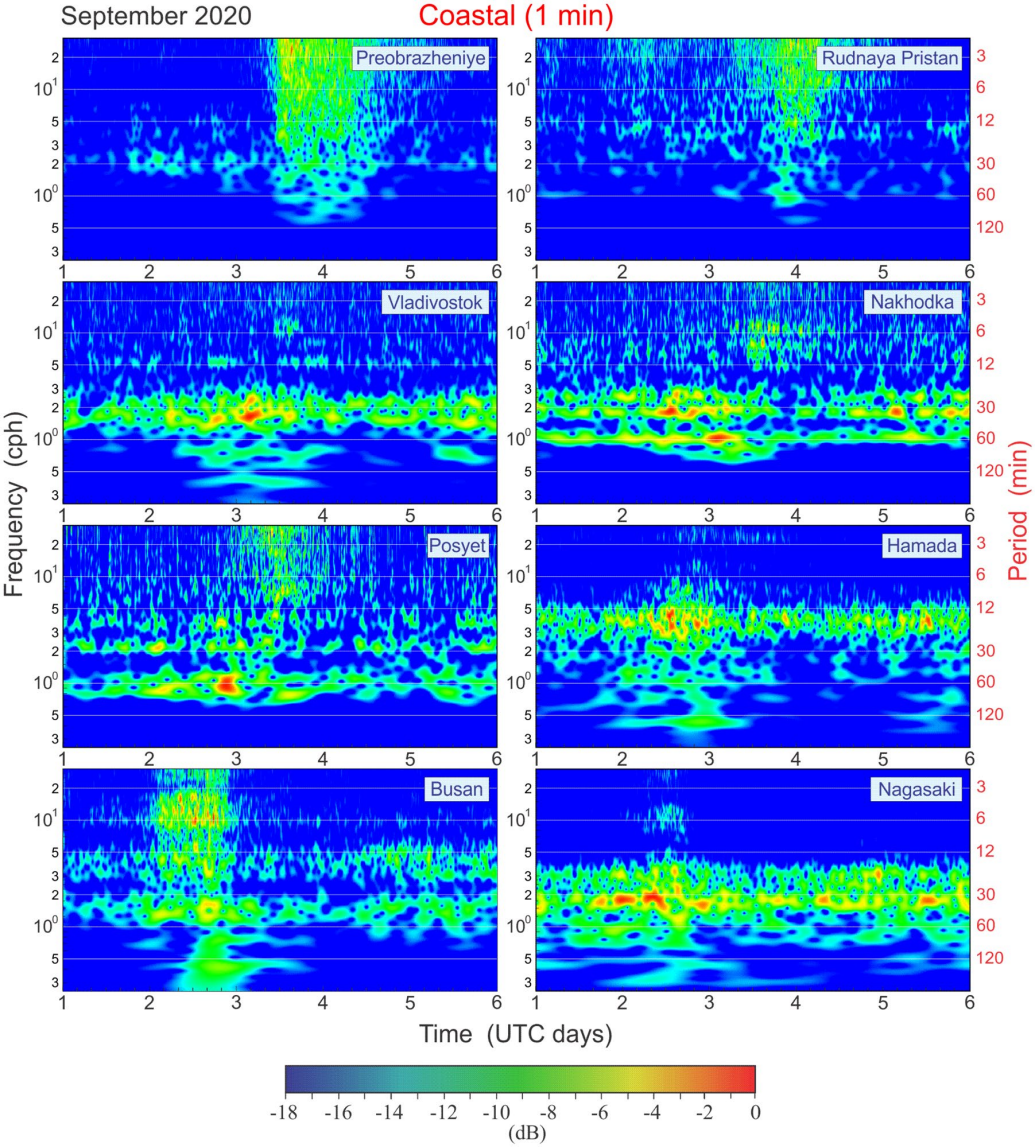
Frequency–time plots (*f–t* diagrams) for the 1–6 September 2020 residual and high-pass filtered sea level records during the passage of Typhoon Maysak for eight tide gauges on the coasts of Japan, South Korea, and Russia.

Wavelet and spectral analyses were used to quantify the magnitudes of the observed oscillations, their frequency content, and transformation (see section “Data and methods” for details). The frequency–time (*f–t*) diagrams, which are similar to wavelet plots^10^, enabled us to examine the evolution of sea level oscillations as function of frequency *f* and time *t* and to display nonstationary effects in the recorded oscillations. Figure 4 shows the *f–t* diagrams for eight tide gauge records for the frequency band of 0.25–30 cph (cycles per hour), i.e. for the periods from 4 h to 2 min.

Narrow frequency bands of significantly amplified energy are evident at most sites. At Vladivostok, Nakhodka, Posyet, and Nagasaki, amplification occurred for period of 30–40 min, at Hamada at a period of 20 min, and at Busan at periods of about 20 and 50 min (Fig. 4). The same amplified bands are clearly seen in the background oscillations prior to the typhoon's arrival. The high stability and persistent character of the “band oscillations” during the entire period examined are indicative of resonant oscillations. Most likely, these marked bands are associated with the eigen frequencies (i.e., local *seiches*) of the respective sites. For a few hours around the core time of the typhoon activity, we can see dramatic amplification of these resonant oscillations and, at the same time, active generation of broad-band oscillations occupying almost the entire frequency band. After the typhoon’s passage, the broad-band oscillations rapidly decayed, while the narrow-band oscillations remained ringing for considerable time.

It is evident in Fig. 4 that sea level oscillations at Russian stations Preobrazheniye and Rudnaya Pristan had a significantly different character than those at the six other sites shown in this figure. We see a “cloud” of typhoon-generated oscillations occupying the entire frequency band from approximately 2–30 cph, wherein spectral energy is evidently cascading from high to low frequencies. This is typical for IG-waves^8^; the main energy source for these waves are highly energetic storm waves, i.e. the energy is transferring from small-scale to larger-scale processes, mimicking the effect of the “negative viscosity”^11^.

In general, the *f–t* analysis demonstrates the evident sea level response to the propagating typhoon and the high importance of the local resonant properties of individual harbours in the formation of hazardous floods, including destructive meteotsunamis^12,13^. Storm-related IG-waves are another important factor amplifying high-frequency oscillations and enhancing the destructive effects of coastal floods^3^.

Spectral analysis was used to provide further insight into the structure of SL oscillations at various stations and for the approximate estimation of the relative contribution of the storm surge, seiche, and IG-wave components (see details and chosen parameters in “Data and methods”). Two observation periods were selected: (1) the 8-day period of 20–28 August 2020 corresponding to background sea level variations before the event; and (2) the 1-day periods during 2–3 September for the South Korean and Japanese stations and 3–4 September for the Russian stations for sea level fluctuations during the event.

**Figure 5:**
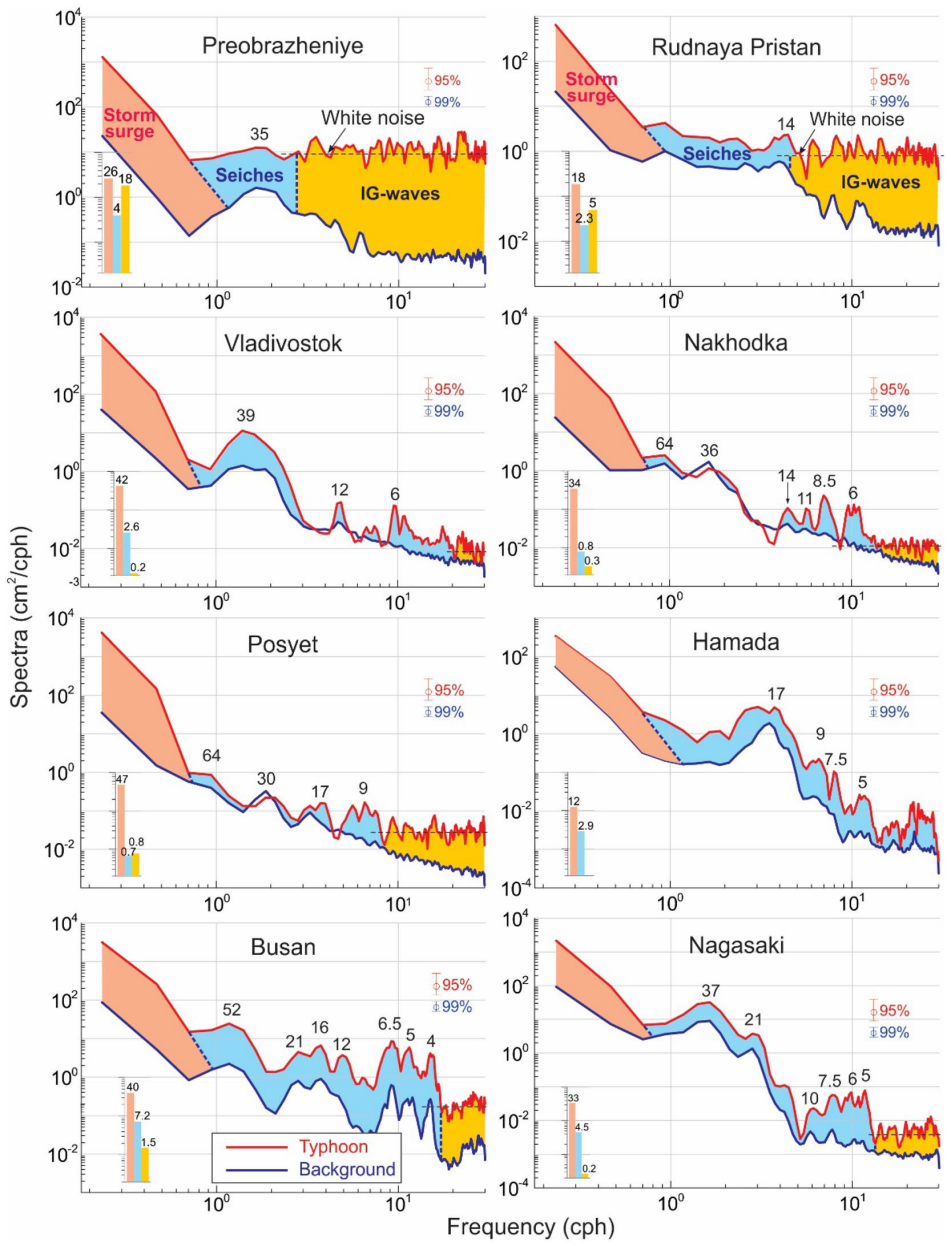
Spectra of background (pre-typhoon) and 2020 Typhoon Maysak sea level oscillations for eight 1-min tide gauge records from the coasts of Japan, South Korea, and Russia. Periods (in min) of the main spectral peaks are indicated. The 95% confidence level applies to the typhoon spectra, the 99% confidence level to the background spectra. The *shaded areas* denote the energy of three types of typhoon-generated oscillations: (1) Storm surge (peach), (2) storm seiches/meteotsunamis (light blue), and (3) infragravity (IG) waves (dark yellow); the bars in the left corners of each plot show the corresponding variances (in cm^2^).

Most of the spectra (Fig. 5) show specific peaks that are the same for the background and event spectra, indicating that the peaks are related either to the main eigen modes of the bays/harbours or to resonant features of the adjacent shelf. In general, the results of the spectral analysis allow us to separate all stations into three groups:

1. “*Resonant*” (*Monochromatic*) stations: these sites have one dominant spectral peak, indicating a high *Q*-factor and, hence, strong resonant amplification of incoming waves^8,14^. As per the terminology of Šepić and Rabinovich^15^, these are “hot spots” that include Nagasaki, which has a prominent spectral peak at 37 min associated with the fundamental mode of Nagasaki Bay. This bay is known for frequent destructive meteotsunamis, locally known as ‘*abiki*’, that can reach trough-to-crest heights of almost 5 m^12,13^. Other sites with sharp spectral peaks and amplified seiches are Hamada (17 min) and Vladivostok (39 min) (Fig. 5).
2. “*Multi-resonant*” (*Polychromatic*) sites. These locations have two, three or more spectral peaks of comparable heights. Nakhodka is characterized by low-frequency (LF) peaks with periods of 64 and 36 min, but also has a number of higher-frequency peaks at 14, 11, 8.5, and 6 min. Similarly, Posyet has two LF peaks—64 and 30 min—and two HF peaks of 17 and 9 min. A large number of peaks are observed at Busan: 52, 21, 16, 12, 6.5, 5 and 4 min.
3. “*White-noise*” sites: These include Russian stations Preobrazheniye and Rudnaya Pristan, where there are no significant spectral peaks, except a minor peak with a period of 35 min at the first station and a period of 14 min at the second (the corresponding frequency bands are also visible in the *f–t* diagrams, Fig. 4). The uniform spectra at these stations are characteristic of *white noise* and are typical of open-ocean coasts that are strongly affected by storm waves, swell, and IG-waves^8,16^.

## Discussion

Typhoons are tropical cyclones (TC) that develop over the Northwestern Pacific between 180° and 100°E. This region is the most active TC-generating basin on Earth, accounting for > 30% of the annual number of tropical cyclones around the globe^1,2^. Typhoons strongly affect the southern Sea of Japan, including the coasts of Japan, Korea and the Primorye Region of Russia; most of them occur in August and September^2,17,18^. The passage of Typhoon Maysak in September 2020, led to numerous fatalities and spawned a large number of other natural hazards, including hurricane-force winds, heavy rainfall and mudslides. One of the most destructive effects of the typhoon passage was landfall floods that caused severe damage to moored ships, harbour facilities and other coastal infrastructure. The present study indicate that these destructive effects were strongly amplified by the superposition of three hazardous meteorologically-induced sea level components: (1) Storm surge; (2) storm seiches; and (3) infragravity waves. The cumulative effect of these components is evident in the tide gauge records (Fig. 3a, Table 1). At the same time, these records reveal markedly dissimilar characteristics of typhoon-induced sea level oscillations at various stations and significant differences in the relative contributions of the three components to the observed total flood height.

*Storm surge* is a low-frequency process with typical periods from a few hours to several days. De-tiding and low-pass filtering with a 3-h window allowed us to isolate this component (Fig. 3a) and estimate its primary statistical characteristics, in particular, surge height relative to mean sea level (MSL) (Table 1). Storm surge is commonly considered the main factor responsible for devastating coastal flooding^7,19^. However, high-frequency sea level oscillations, generated by typhoon/hurricane passages, can substantially increase the flood height and be the reason of other damaging effects, including highly intense currents^3^. In the present case, Maysak forced HF long waves that enhanced the total flood height at all stations, most of all at Busan—from 79 to 115 cm and at Preobrazheniye from 32 to 124 cm (Table 1).

As indicated above, storm-induced HF sea level oscillations consist of two principal components: (1) Storm seiches; and (2) IG-waves. These two types of waves have entirely different forcing and generation mechanisms. Seiches are standing waves in inner basins (*eigen oscillations*) and on the shelf (*shelf seiches*) that have a resonant nature and are mainly generated by atmospheric pressure disturbances and squall lines^8^. Extreme seiches are commonly associated with *meteotsunamis*^13,20,21^, while infragravity waves are formed through the nonlinear interaction of wind waves and swell; the most intensive IG-wave formation occurs in the coastal, wave-breaking zone, where these waves are known as *surf beats*^9,16^. Typical periods of seiches are from a few minutes to a few hours, while conventional periods of IG-waves are an order of magnitude shorter (30–300 s). However, strong storms produce intense energy transfer from high (wind-wave) frequencies to low frequencies; so, IG periods can reach 30 min and longer^11^. This means that the frequency bands of storm-generated seiches and IG-waves partly overlap, impeding their separation in our analyses. Fortunately, the spectral properties of the two processes are quite different, helping us distinguish the IG-waves from atmospherically-induced seiches.

Figure 5 shows the separation of the three types of sea level components in the spectral domain. The LF (*surge*) spectral component is typified by a rapid decrease of the spectra up to a frequency of around 0.8 cph (period of ~ 75 min), while the *seiche* component is distinguished by a number of resonant spectral peaks associated with eigen frequencies of the corresponding basins. The IG spectral component resembles white noise. The spectral estimates show the principal role of the IG-waves at Preobrazheniye, where they had amplitudes of up to 90 cm, thereby exceeding the surge height by a factor of roughly three. The IG-waves are also important at Rudnaya Pristan and are noticeable at Posyet, but play a negligible role at all other examined sites (Fig. 5).

The question that arises from our findings is *Why was the sea-level response to the typhoon passage so different at different sites?* The most obvious conclusion is that the response was determined by the speed and the trajectory of the typhoon relative to a given site, and by shelf and near-coast bathymetry and coastal geometry. Clearly, the storm surge effect was the strongest over the southern coast of South Korea, i.e. at those locations where the typhoon made a direct landfall, implying that air pressure was lowest and onshore winds were the most powerful. Nevertheless, similar (or even higher!) flood heights were observed at Russian stations Preobrazheniye and Rudnaya Pristan, which were further away from the typhoon’s main track and where the storm surge effect was much less pronounced than the effect of IG-waves.

It is well known that destructive storm surges normally occur in regions with an extensive shallow-water shelf, such as the Bay of Bengal, the northeastern Gulf of Mexico^7^ or the Gulf of Finland in the Baltic Sea^22^. Similarly, we found that the topographic factors control the sea-level reaction to the passing typhoon and the spectral properties of generated HF waves. Analyses of specific coastal features at tide gauge sites affected by Typhoon Maysak^12,23,24^, as well as some other sites around the world^8,25^, have demonstrated that:

- *Monochromatic* seiches are observed in long narrow inlets (fjords) or in narrow-mouth bays and well-protected harbours. The fundamental (“pumping”) mode strongly prevails in such basins and amplifies during typhoon events (cf. Nagasaki and Hamada).
- *Polychromatic* (multi-mode) seiches typical occur in bays and harbours with relatively wide mouths and in poorly sheltered harbours (e.g., Posyet and Busan).
- *Infragravity waves* (*surf beats*) are predominant at sites located at coasts that are open to incoming wind waves and swell (e.g., at Preobrazheniye and Rudnaya Pristan).

These results clearly indicate that the differences in sea level response to Typhoon Maysak we have observed are explained by the specific properties of the various sites. Potentially, these properties can be taken into account and in the future be used for prediction and warning.

**Figure 6:**
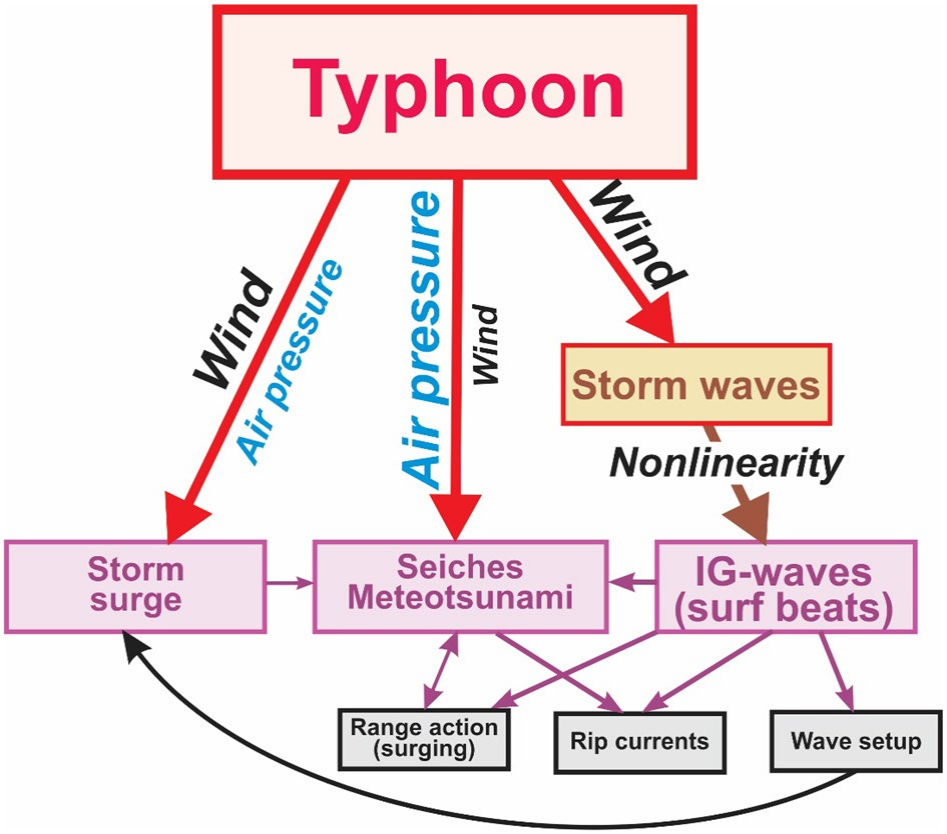
A sketch showing the various types of hazardous sea level oscillations and related phenomena generated by a typhoon.

Another crucial question is *What are the specific typhoon factors that are responsible for coastal floods and hazardous sea level oscillations?* Typhoons commonly bring very heavy rains and associated damaging floods. However, these floods mostly occur within *inland* regions. The two main factors that produce *coastal* sea level oscillations and associated floods are *atmospheric pressure* and *wind.* Figure 6 illustrates the specific forcing and generation mechanisms of the three main components, which are essentially different for the different types of SL oscillations.

- *Storm surges* are produced by strong winds and low air pressure that occur during powerful storms^7^. Long-lasting onshore winds over vast shallow-water shelves are the main cause of ruinous surges.
- *Seiches* are natural (eigen) oscillations in closed or semi-closed basins. They can be induced by various forcing factors and by energy transfer from storm surges. However, the major factor is air pressure disturbances, such as pressure jumps, internal gravity waves and various other pressure perturbations. Seiches can also be caused by strong wind gusts and squalls. All of these features are typical of typhoons. Extreme seiches are known as *meteotsunamis*^13^ and are likely effectively generated by typhoons and hurricanes at specific sites as: (1) they are much more *compact* than extratropical cyclones and their spatial dimensions can be comparable with those of inner basins, where these seiches occur; (2) correspondingly, they have high air pressure gradients, which is a key factor promoting seiche generation; and (3) the typical propagation speed of typhoons/hurricanes (up to 100 km/h) is comparable with the speed of long ocean waves on the shelf corresponding to an effective speed for water depths of ~ 75 m). In contrast, the typical speed of mid-latitude cyclones (of about 40 km/h) is too low for the resonant generation of meteotsunamis, which occurs when these speeds coincide (Proudman resonance^13^).
- Typhoon-induced *infragravity waves* have a two-cycle generation mechanism: (1) intense typhoon winds induce giant storm waves that have periods of 10–20 s and wave heights of more than 20 m; (2) the storm waves in the nearshore surf zone then generate much longer-period oscillations through nonlinear interactions—IG-waves—known in this zone as *surf beats*^6^. Surf beats strongly dominate the velocity field close to the shore and produce a number of specific phenomena^8,16^, including:
  - *Rip currents*—highly intense, narrowly channeled currents directed offshore; during strong typhoons and hurricanes, rip-current speeds can be more than 10 knots (5 m/s). Rip currents, which are closely related to IG-waves^25,26^, are considered as one of the most dangerous natural disasters in the world: in the US, lifeguards rescue tens of thousands of people every year from rip currents. Hazardous rip currents can also be caused by meteotsunamis^27^.
  - *Wave setup*—a sea-level increase in the surf zone due to the transfer of the storm wave-related momentum to the water column; wave setup can add 10–20% to the height of wind-induced storm surge^7^.
  - *Range action* (*surging*)—periodic horizontal water motions observed in many harbours around the world that create strong vessel movement, breaking of mooring lines, fenders, and piles^28–30^. The main generation factor of this phenomenon is strong IG-waves that propagate into the harbour, but it also can be caused by atmospherically induced seiches in the harbour.

Figure 6 shows that a passing typhoon produces several interdependent types of hazardous sea level oscillations and related phenomena. It appears that the cumulative effect of these processes was the main reason for the fatal accidents and severe coastal damage that occurred during Typhoon Maysak. It is evident that aggregate phenomena and oscillations should be examined in combination and that considerable attention should be paid to topographic properties and the coastal geometry of specific sites. This applies to any coastal region impacted by extreme atmospheric processes (typhoons, hurricanes, derecho, deep cyclones, etc.), including Japan, West Australia, the US East Coast, the Gulf of Mexico, and the Atlantic coast of West Europe.

## Data and methods

Our analysis of Typhoon Maysak uses variables from the ERA5 Reanalysis Dataset^31^, specifically: the 10-m *U* and *V* wind components, mean sea level pressure (MSLP), and maximum 3-s 10-m elevation wind gusts during the last hour. The spatial resolution of the ERA5 Reanalysis Dataset is 0.25° × 0.25°, and the time step is 1 h. The ERA5 data were downloaded from the CDS API and processed using MATLAB. The satellite images of Typhoon Maysak were downloaded from the VentuSky (http://www.ventusky.com) website and edited using CorelDRAW.

The South Korean meteorological data for stations Jeju Seongsan, Geojedo, Busan, Ulleang, Donghae, and Sokcho (atmospheric pressure and wind) have a 1-min sampling interval and were downloaded from the Korean Hydrographic and Oceanographic Agency data portal (http://www.khoa.go.kr/oceangrid/khoa/koofs.do). The Russian (Vladivostok and Nakhodka) and Japanese (Kumamoto and Tokoji) meteorological data have 5-min time steps and were downloaded from the Weather Underground Wundermap (https://www.wunderground.com/wundermap) data portal. The resolution of the air pressure measurements is 0.1 hPa for the South Korean records and 0.1 inches Hg (~ 0.3 hPa) for the Russian and Japanese records. The resolution of the wind speed measurements is 0.1 m/s (~ 0.4 km/h) for the South Korean data and 0.1 mph (~ 0.2 km/h) for the Russian and Japanese data. In order to unify the meteorological data, we low-pass filtered the Korean 1-min data with a 10-min Kaiser–Bessel window and subsampled the output at a 5-min time step. The processing of meteorological records included quality control and high-pass filtering with a 3-h Kaiser–Bessel window^10^.

The Japanese and South Korean sea level data were downloaded from the Sea Level Station Monitoring Facility (UNESCO/IOC, http://www.ioc-sealevelmonitoring.org/), while the Russian data were from http://www.rtws.ru/sea-level/. All records have a 1-min sampling, with height resolutions of 1 cm for the Russian and South Korea records and 0.1 cm for the Japanese records.

The data analysis procedures of sea levels were similar to those used by^3,15,32^ to examine sea level oscillations generated by atmospheric processes. The data underwent quality control to remove gaps, spikes, and outliers. The records for all stations were then de-tided using the least squares method and the resulting residual series were used for further analyses. To examine the spectral properties of sea level oscillations, we separated the records into two parts: (a) the 8-day pre-typhoon period, which was used to analyze background oscillations, and (b) the 23.5 h period following the typhoon arrival, which was used for the analysis of typhoon-generated waves.

Our spectral analysis procedure is similar to that described by Thomson and Emery^10^. To improve the spectral estimates, we used a Kaiser–Bessel spectral window with half-window overlaps prior to the Fourier transform. The length of the window was chosen to be *N* = 256 min, yielding *ν* = 178 degrees of freedom for the background spectra and *ν* = 20 for the event spectra; the spectral resolution was Δ*f* = 0.234 cph.

## Data Availability

Sea level data is available at the Sea Level Station Monitoring Facility of IOC (UNESCO) (http://www.ioc-sealevelmonitoring.org/index.php), the Federal Service for Hydrometeorology and Environmental Monitoring of Russia (http://rtws.ru/sea-level/), and the Korean Hydrographic and Oceanographic Agency (http://www.khoa.go.kr/oceangrid/khoa/koofs.do). Atmospheric data is available at the Weather Underground Wundermap (https://www.wunderground.com/wundermap) and the Korean Hydrographic and Oceanographic Agency (http://www.khoa.go.kr/oceangrid/khoa/koofs.do). ERA5 Reanalysis data is available at Climate Data Store (https://cds.climate.copernicus.eu/#!/home). The satellite images are available at Ventusky website (http://www.ventusky.com).
